# Application of Fluorescence Dye in Combination with Methylene Blue for Axillary Reverse Mapping in Patients with Modified Radical Mastectomy for Breast Cancer

**DOI:** 10.1155/2022/2305542

**Published:** 2022-05-14

**Authors:** Haiyi Wang, Baochao Wu, Zheng Wang

**Affiliations:** ^1^Department of Thyroid and Breast Surgery, Tongling people's hospital, Tongling, Anhui 244009, China; ^2^Department of Thyroid and Breast Surgery, Tongling Branch of the First Affiliated Hospital of University of Science and Technology of China, Tongling, Anhui 244009, China; ^3^Department of Vascular Surgery, Tongling Branch of the First Affiliated Hospital of University of Science and Technology of China, Anhui 244009, China

## Abstract

**Background:**

Axillary reverse mapping (ARM) is a novel intraoperative technique developed in recent years. This study was aimed at determining the effect of combined use of fluorescence dye and methylene blue, as well as its feasibility of ARM in patients with breast cancers who undergo modified radical mastectomy.

**Method:**

From January 2016 to June 2017, 46 patients with primary breast cancer at stage I-IV (Tis-T3, N0-N3, and M0) who received modified radical mastectomy were enrolled in this study. The exclusion criteria included preoperative radiotherapy/chemotherapy or bilateral disease. Patients were divided into 2 groups: methylene blue group (22 patients, 47.8%) and methylene blue+indocyanine green (ICG) group (24 patients, 52.2%). ARM was performed before surgery.

**Results:**

The overall visualization rate of ARM nodes was 80.4% (37/46). The visualization rate was significantly increased in methylene blue+ICG group (91.67%, 22/24) than that in methylene blue group (63.64%, 14/22) (*P* = 0.032). There was a statistical difference of visualization rate between clinical groups of N0-N1 and N3-N4 (*t* = 2.431, *P* = 0.19 < 0.05). Although there was no significant difference found in the total drainage volume and arm perimeter of patients between the two groups (*P* > 0.05), the harden diameter of the injection site in methylene blue+ICG group was significantly longer than that in methylene blue group (*P* < 0.05). Among the 44 patients with different molecular profiling of breast cancer, there was no significant difference of visualization rate of ARM nodes in luminal A group (100%,5/5), luminal B group (75.0%, 18/24, *P* = 0.21), HER2 group (75.0%, 6/8, *P* = 0.22), and basal-like group (85.7%, 6/7, *P* = 0.37).

**Conclusions:**

ARM using methylene blue+ICG presented greater identification rate than that with methylene blue alone, especially in patients with more invasive breast cancer. Our finding offers support for the improvement of ARM in future breast cancer management.

## 1. Introduction

Although new markers for oncogene expression were identified and showed promise in guiding treatment strategies for breast cancer, the condition of the axillary lymph nodes affects treatment and remains one of the most important prognostic factors of outcome [1]. Currently, axillary lymph node dissection (ALND) represents the standard treatment for patients with metastatic axillary lymph nodes. However, ALND has high incidence of upper extremity lymphedema, ranging from 7% to 77%, and arm/shoulder dysfunction, paresthesia, and discomfort are found after the surgery [2]. Sentinel lymph node biopsy (SLNB) is recommended for patients with clinically node-negative axilla at surgery, which significantly decreases surgical complications compared with ALND [3, 4]. Nevertheless, several reports on clinical trial have shown that the rate of lymphedema was approximately 7% with SLNB alone [5].

Axillary reverse mapping (ARM) is a technique described initially by Thompson et al. [6] and Nos et al. [7] in 2007, to delineate the lymph nodes (named ARM nodes) and lymphatic vessels of the upper limb during the axillary surgery in breast cancer patients. In this technique, the ARM nodes can be spotted by injecting a tracer into the upper arm prior to surgery, similar to the identification of the sentinel lymph node (SLN) that drains the breast [6]. The ultimate aim of ARM is to limit the occurrence of arm lymphedema based on the hypothesis that the ARM procedure actually identifies a different lymphatic pathway coming from the arm [5]. The addition of ARM to ALND or SLNB may reduce lymphedema of the upper extremity by preserving upper extremity drainage. So far, three visualization methods have been used in ARM, such as radioisotopes, fluorescence dye, and blue dye. The blue dye is the most commonly used way, whereas only five studies have reported the application of fluorescent dye indocyanine green (ICG) so far. In this study, the effect of combined use of blue dye and ICG is assessed during the modified radical mastectomy on patients with breast cancers.

## 2. Patients and Methods

### 2.1. Study Cohort

From January 2016 to June 2017, 46 patients who underwent modified radical mastectomy were enrolled in this study. They were diagnosed as primary breast cancer at clinical stage I-IV (Tis-T3, N0-N3, and M0) in our hospital. Those with preoperative radiotherapy/chemotherapy or bilateral disease were excluded. Ethical approval for this study was provided by the local ethical committee of our hospital. The median age of the study cohort was 50 years old (range 34-77). Informed consent was obtained from each patient prior to surgery.

Patients were divided into 2 groups. The control group (methylene blue group) consisted of patients receiving ARM using methylene blue alone (22 patients, 47.8%). The experiment group (methylene blue+ICG group) received the combination of ICG and methylene blue (24 patients, 52.2%). In the methylene blue group, 2.0 ml of methylene blue (Jumpcan Pharmaceutical Group Co., Ltd., Jiangsu, China, medicine number: H32024827) was subcutaneously injected into the upper inner arm. Then, the injection site was massaged, and the arm was raised for 10 min until the blue lymphatic vessels were observed in the upper extremity. Similar procedure was used in the methylene blue+ICG group for injection of 2.0 ml methylene blue plus 5 mg ICG (Tianyi Biopharmaceutical Co., Ltd., Liaoning, China, medicine number:H20045514). The clinical characteristics of the study cohort were summarized in Table 1.

### 2.2. Axillary Reverse Mapping

**Figure 1:**
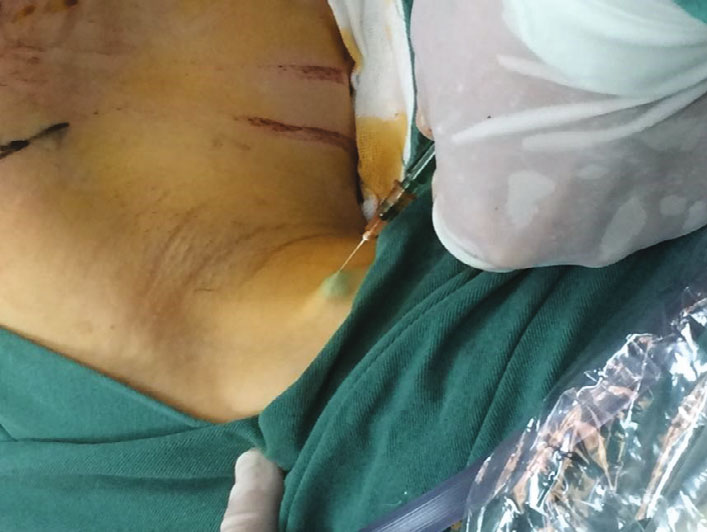
Subcutaneous injection of ICG into the ipsilateral upper inner arm.

The axillary reverse mapping (ARM) technique is developed for identifying and preserving lymphatic drainage from the arm during ALND. ARM was performed before the surgery. Before ALND, 2.0 ml of methylene blue with or without 5 mg of ICG in 2 ml of normal saline (NS) was subcutaneously injected into the ipsilateral upper inner arm of experiment or control group, respectively (Figure 1). The injection site was massaged gently for 10 min after the injection.

### 2.3. Axillary Lymph Node Dissection

**Figure 2:**
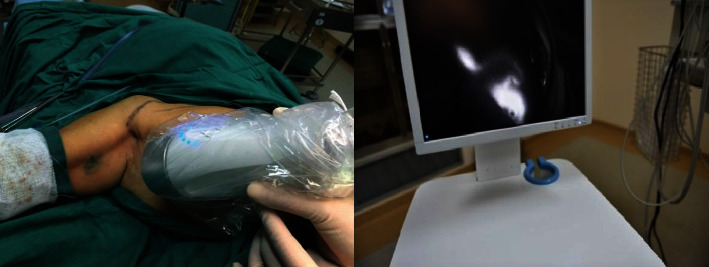
Intraoperative fluorescence imaging for ARM nodes.

**Figure 3:**
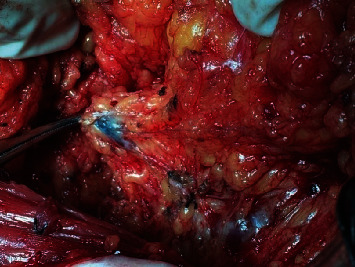
ARM lymph nodes dyed with blue dye.

The primary ALND was indicated based on enlarged axillary lymph nodes by imaging assessment. ARM node labeled with ICG was observed by a fluorescence imaging camera (Langfang Mingde Biological Medicine Technology Co., Ltd., Hebei, China), while methylene blue was viewed with the naked eyes (Figures 2 and 3). Intraoperative identification rates of ARM nodes as well as the metastasis rates of ARM nodes were then evaluated. During the surgery, ARM nodes were first identified and removed separately and then examined by pathologic tests.

### 2.4. Pathological Examination

All patients were diagnosed with the evaluation of postoperative TNM stage based on the revised TNM staging system adopted by NCCN Clinical Practice Guidelines in Oncology: Breast Cancer (Version 1.2016).

Molecular profiling of breast cancer was performed for each patient and identified as luminal A, luminal B, basal-like, and overexpressed HER2 type and the estrogen/progesterone receptor (ER/PR) status as well as HER2/neu status.

### 2.5. Statistical Analysis

Statistical analysis was conducted by using SPSS, version 17.0. The chi-square and *T* test analysis were used to estimate intergroup differences. A *P* value < 0.05 was considered to be statistically different.

## 3. Results

### 3.1. Successful Rate of Methylene Blue or Methylene Blue+ICG Dyeing

The overall successful dyeing rate of ARM nodes was 80.4% (36/46). 22 patients received dyeing of methylene blue alone, while 24 patients were given treatment of methylene blue+ICG. Visualization rate was significantly higher in the methylene blue+ICG (91.67%, 22/24) group than that in the methylene blue alone group (63.64%, 14/22) (*P* = 0.032). Moreover, among the 24 patients in the methylene blue+ICG group, the diagnosis result on lymph node showed that 20 (83.31%) cases belonged to clinical N0-N1 and 4 (16.67%) cases to clinical N3-N4, while in the methylene blue group, 16 (76.19%) belonged to clinical N0-N1 and 5 (23.81%) to clinical N3-N4. The differences of clinical N0-N1 stage and N3-N4 stage between methylene blue group and methylene blue+ICG group presented statistically significant (*t* = 2.431, *P* = 0.19).

### 3.2. Postoperative Drainage Volume

The total drainage volume includes parasternal drainage volume and axillary drainage volume. We observed that the total drainage volume in the methylene blue+ICG group was slightly lower than that of the methylene blue group, though the difference was insignificant (*P* > 0.05) (Table 2).

### 3.3. Postoperative Perimeter of Limb

**Figure 4:**
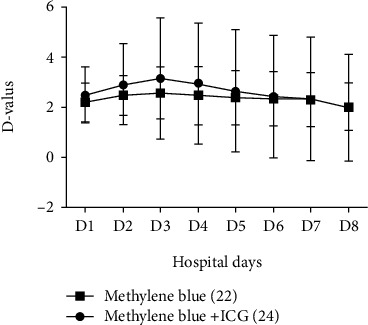
The perimeter difference between limb with operation and healthy one.

There was no significant difference in the total hospital days required for patients in these two groups (*P* > 0.05). The difference of limb perimeter between the patients in the group of methylene blue+ICG was to some extent higher than that in methylene blue group from day 1 to day 6. At days 7 and 8, perimeter difference of the two groups tended to be consistent (Figure 4 and Table 3).

### 3.4. Postoperative Harden Diameter of the Injection Site

**Figure 5:**
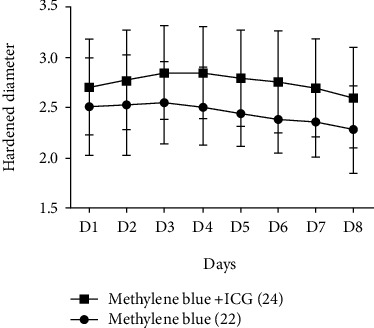
The harden diameter of the injection site after surgery.

From day 1 to day 8, the harden diameter, which is the blue tattooing that occurs at the injection site, exhibited the large tendency in the methylene blue+ICG group compared to that of methylene blue group. Of note, from day 3 to day 8, there was significant difference of harden diameter of the injection site between the two groups after the surgery (*P* < 0.05) (Figure 5 and Table 4).

### 3.5. Positive Rate of Lymph Node

In the methylene blue group, the number of positive axillary lymph node dissections was 11 (11/22,50%), and in the methylene blue+ ICG group, the number was 11 (11/24,45.83%), indicating that the positive rate of lymph node in methylene blue+ICG group was slightly lower than that of methylene blue group (Table 5).

## 4. Discussion

The concept underlying the ARM technique assumes that lymphatics draining the upper extremity are not involved in the metastasis of the primary breast tumor [5, 6]. However, those studies also reported the incidence of metastatic tumor in the ARM nodes is 14%-43%. Currently, most of the studies on ARM technique focus on the feasibility of ARM and limit the incidence of lymphedema through preserving the ARM node/lymphatics, but not the safety. Therefore, in this study, we aim to improve the ARM procedure with satisfactory safety by using combined dyeing of ICG and methylene blue, so as to increase the identification rate of the ARM nodes.

Based on the previous finding of the presence of metastasis in the ARM nodes, our data indicated 17.3% of the metastatic rate in the ARM nodes, which is similar to that of the earlier results of 13% by Kumar et al. [8] and 14% by Nos et al. [7]. Kumar et al. [8] had reported that parameters such as location, consistency, and intraoperative FNAC of the ARM node were reliable to predict the involvement of the ARM node with metastasis.

The initial studies on ARM in 2007 marked a blue dye applied in mapping axillary upper extremity lymph nodes and lymphatics. In accordance with the initial reports, blue dye has been generally used to delineate the axillary upper extremity lymph drainage. The result of visualization rates in the axilla of ARM lymph nodes during ALND ranged from 39% to 90% while of ARM lymphatics from 47% to 86% [9]. In addition, the visualization rates during SLNB of ARM lymphatics ranged from 20% to 47% [10, 11]. Alternatively, a pilot study using the fluorescent dye ICG described that the visualization rates of the ARM lymph nodes and the ARM lymphatics in the ALND field were 88 and 63%, respectively [12]. In the same study, visualization of the ARM lymph nodes and the ARM lymphatics during SLNB was only 43 and 19%, respectively.

In our study, the identification rate of the ARM node was 80.4%, which is similar to 61% by Thompson et al. [6], 75% by Kumar et al. [8], and 71% by Nos et al. [7]. Our data on visualization rate in methylene blue+ICG group (91.67%, 22/24) was significantly higher than that in methylene blue alone group (63.64%, 14/22). Particularly, there are significant differences of visualization rate between the two groups at stage of clinical N0-N1 and N3-N4 (*t* = 2.431, *P* = 0.19 < 0.05). These findings suggested that methylene blue+ICG could improve identification rate of ARM node, especially for patients with more invasive breast cancer.

Although there were no statistical differences in the total drainage volume and perimeter difference between ICG+methylene blue group and methylene blue alone group, the harden diameter of the injection site was significantly increased in methylene blue+ICG group than that in methylene blue alone group. These results indicate the feasibility of ARM using fluorescence+blue dye over blue dye alone in the Chinese breast cancers patients who received modified radical mastectomy.

## 5. Conclusions

ARM using methylene blue+ICG presents greater identification rate of ARM node than that with methylene blue alone, which provides a potential tool for the surgical therapy towards patients with invasive breast cancer.

## Figures and Tables

**Table 1:** Clinicopathological characteristics of patients.

Characteristics	Methylene blue+ICG (24)	Methylene blue (22)	*P*	Characteristics	Methylene blue+ICG (24)	Methylene blue (22)	*P*
Median age	48 (34~77)	51 (37~68)					
HER2			0.375	Estrogen receptor status			0.072
Negative	15	10		Negative	12	5	
Positive	9	12		Positive	12	17	
Histologic type			0.987	Progesterone receptor status			0.085
Invasive ductal	15	15		Negative	14	7	
Invasive lobular	5	6		Positive	10	15	
Mix	4	1		P53			0.139
Clinical staging			0.421	Negative	9	4	
I	0	3		Positive	6	11	
II	11	11		VEGF			0.433
III	3	1		Negative	3	6	
IV	0	0		Positive	12	10	
I~II	3	2		E-cad			1
II~III	7	5		Negative	0	1	
Topo II			0.484	Positive	16	15	
Negative	1	0		Clinical T stage			0.285
Positive	14	16		T1	13	7	
GST-*π*				T2	11	12	
Negative	6	7	0.92	T3	0	1	
Positive	9	9		Clinical N stage			0.64
p170				N0	10	10	
Negative	10	9		N1	10	6	
Positive	5	7	0.716	N2	3	2	
Ki67 score				N3	1	3	
<30%	7	12					
30%~70%	13	8					
70%<	4	2	0.259				

**Table 2:** Postoperative drainage volume.

Drainage volume	Methylene blue+ICG (24)	Methylene blue (22)	*t*	*P*
Total drainage volume	573.9 ± 286.43	590.5 ± 265.64	0.189	0.849
Parasternal drainage volume	389.4 ± 222.87	353.6 ± 182.12	0.551	0.584
Axillary drainage volume	184.5 ± 152.42	236.8 ± 138.27	1.135	0.258

**Table 3:** Perimeter difference between suffered limb and healthy one.

Days	Perimeter difference		*t*	*P*
	Methylene blue+ICG (24)	Methylene blue (22)		
D1	2.513 ± 1.121	2.24 ± 0.755	0.872	0.366
D2	2.93 ± 1.62	2.5 ± 0.791	1.041	0.304
D3	3.169 ± 2.424	2.6 ± 1.043	1.013	0.358
D4	2.965 ± 2.412	2.488 ± 1.166	0.796	0.447
D5	2.678 ± 2.445	2.405 ± 1.09	0.439	0.663
D6	2.456 ± 2.45	2.378 ± 1.079	0.127	0.9
D7	2.357 ± 2.468	2.328 ± 1.087	0.46	0.96
D8	2.001 ± 2.132	2.044 ± 0.945	0.066	0.948
Total hospital days	11.434 ± 3.553	11.833 ± 3.148	0.374	0.710

**Table 4:** Postoperatively harden diameter of the injection site.

Hospital days	Harden diameter		*t*	*P*
	Methylene blue+ICG (24)	Methylene blue (22)		
D1	2.709 ± 0.477	2.5167 ± 0.484	1.27	0.211
D2	2.783 ± 0.496	2.533 ± 0.501	1.589	0.12
D3	2.856 ± 0.463	2.555 ± 0.409	2.172	0.036
D4	2.852 ± 0.459	2.511 ± 0.376	2.552	0.015
D5	2.8 ± 0.479	2.45 ± 0.326	2.655	0.011
D6	2.761 ± 0.509	2.394 ± 0.336	2.663	0.012
D7	2.7 ± 0.486	2.368 ± 0.356	2.438	0.019
D8	2.604 ± 0.501	2.288 ± 0.434	2.119	0.041

**Table 5:** The positive and negative lymph nodes.

Groups	Lymph node positive	Lymph node negative
methylene blue+ICG(24)	11 (45.83%)	13 (54.17%)
Methylene blue(22)	11 (50%)	11 (50%)

## Data Availability

Emails could be sent to the address below to obtain the shared data: wangzdr@yeah.net
